# Validation of ATP bioluminescence as a tool to assess antimicrobial effects of mouthrinses in an in vitro subgingival-biofilm model

**DOI:** 10.4317/medoral.18376

**Published:** 2012-12-10

**Authors:** María C. Sánchez, Arancha Llama-Palacios, María J. Marín, Elena Figuero, Rubén León, Vanessa Blanc, David Herrera, Mariano Sanz

**Affiliations:** 1 ETEP (Aetiology and Therapeutics of Periodontal Diseases) Research Group, University Complutense of Madrid, Spain; 2 Dentaid SA, Barcelona, Spain

## Abstract

Objectives. The aim of this investigation was to evaluate whether the adenosine triphosphate (ATP) bioluminescence method is an appropriate tool to assess the efficacy of antiseptic mouthrinses in terms of quantitative reductions of total viable microbial counts in mixed biofilm populations in vitro. Study Design. Three mouthrinses, containing respectively, chlorhexidine and cetylpyridinium chloride (CHX/CPC), essential oils (EO) and amine fluoride/stannous fluoride (AFSF), as well as Phosphate Buffered Saline (PBS) used as control, were tested in an in vitro static biofilm model by ATP bioluminescence and compared to culture method. Biofilms were grown on saliva-coated hydroxyapatite disks for 72 hours and then exposed for 1 minute to the mouthrinse or control by immersion. The antibacterial effect of the rinses was tested by analysis of variance. The reliability of the ATP bioluminescence method was assessed by calculating the Pearson correlation coefficients when compared to the viable cell counts obtained by culture. Results. Using ATP bioluminescence, the antimicrobial activity of the tested mouthrinses was demonstrated when compared to the PBS control. The ATP bioluminescence values were significantly correlated (0.769, p<0.001) to the viable cell counts. CHX/CPC and AFSF showed similar antimicrobial activity, although AFSF had a less homogeneous effect, being both more effective than the EO rinse. Conclusion. ATP bioluminescence viability testing may be considered a useful tool to assess the in vitro efficacy of antibacterial compounds. In the proposed model, CHX/CPC and AFSF containing mouthrinses demonstrated superior antimicrobial activity, as compared to EO rinses, in a multispecies biofilm model.

** Key words:**Biofilm, ATP bioluminescence,mouthrinse, essential oils, chlorhexidine, amine fluoride/stannous fluoride.

## Introduction

The effective control of the dental plaque is the key in the prevention of periodontal diseases. Several studies have demonstrated that the mechanical removal of the supragingival plaque through effective oral hygiene practices prevents or reverses the inflammatory status of the gingival tissues (1-3). Epidemiological data indicates, however, that most individuals do not control plaque accumulation to a sufficient extent to prevent or control the occurrence of this condition, probably due to lack of motivation or skills, or both (3,4). In order to overcome this hindrance, antimicrobial oral hygiene products have been investigated in their efficacy to additionally reduce plaque and gingivitis when used daily as adjuncts to mechanical plaque control. Since human dental plaque is a dynamic and complex biofilm where bacteria from saliva are adhered to tooth surfaces embedded in a matrix of extracellular polymers (5), the efficacy of these antimicrobials must be tested within these environments rather than in planktonic status, due that bacteria in matured biofilms are less susceptible to antimicrobial agents because of several physical and biological factors that protect the bacterial consortia (6-8).

Several studies have attempted to study the effect of mouthrinses in biofilms*in vitro* (8-12). Traditionally, bacterial counts on agar plates was the method of choice for determination of bacterial viability, although this method has clear limitations, as the relatively long times needed for the colony growth, the differences in the growth media used or the likely growth inhibition by neighbouring cells (13). The use of morphological methods, as Confocal Laser Microscopy (CLSM), are useful to assess the structure and physiology of biofilms, but it does not allow the assessment of changes in the bacterial viability when biofilms are exposed to antiseptic compounds (11). Also, culture-independent molecular methods for identification and quantification of oral bacteria have been extensively developed during recent years, but they are still not widely used in routine laboratories, due to the relatively long persistence of DNA after cell death, in the range between days to 3 weeks, what may overestimate the number of live cells after an antiseptic treatment (14). One possible alternative is the adenosine triphosphate (ATP) bioluminescence method, which has been utilized as a quantitative assay to evaluate viable bacteria in different biological samples, as well as in dental plaque (15-19). This method is based on the activity of the nucleotide ATP as a key element in the energy exchange of all biological systems. ATP serves as the principal immediate donor of energy and it is present in all metabolically active cells, since it links catabolic and anabolic processes. When cells are lysed, the released ATP can be measured by bioluminescence through its reaction with the luciferin-luciferase. This reaction is catalyzed by the enzyme luciferase obtained from the firefly *Photinus pyralis* that uses the chemical energy contained in the ATP molecule to drive the oxidative decarboxylation of luciferin. The MgATP2- converts the luciferin into a form, which is capable of being catalytically oxidized by the luciferase in a high quantum yield chemiluminescent reaction at 562 nm (19). The main advantage of this technique is the provision of a rapid and real-time quantification of viable bacteria, however this method has not been previously utilized to test the antimicrobial efficacy of antiseptic mouthrinses in an *in vitro* complex oral biofilm model.

There are few published *in vitro* biofilm models using a consortium of anaerobic bacteria where antimicrobial compounds can be adequately tested. Our research group has recently developed and tested such an in vitro biofilm model reporting its structure, viability and bacterial kinetics (20). This model uses six bacteria from the subgingival biofilm, containing initial (*Streptococcus oralis and Actinomyces naeslundii*), early (*Veillonella parvula*), secondary (*Fusobacterium nucleatum*), and late colonizers (*Porphyromonas gingivalis and Aggregatibacter actinomycetemcomitans*).

It is, therefore, the main purpose of this investigation evaluate the possibility to use ATP bioluminescence method for rapid quantitative evaluation of viable total oral bacteria in biofilms samples, as compared to standard culture methods, to test the bactericidal efficacy of antiseptic mouthrinses. For this purpose, we used three commercially available antiseptic mouthrinses containing respectively, chlorhexidine digluconate (CHX) and cetyl-pyridinium chloride (CPC), essential oils (EO), and amine fluoride/stannous fluoride (AMSF) in a tested and validated in vitro biofilm model (20).

## Material and Methods

-Bacterial strains and culture conditions

Standard reference strains of *S. oralis CECT 907T, V. parvula* NCTC 11810, *A. naeslundii* ATCC 19039, *F. nucleatum* DMSZ 20482, *A. actinomycetemcomitans* DSMZ 8324 and *P. gingivalis* ATCC 33277 were used. Bacteria were grown on blood agar plates (Blood Agar Oxoid No 2; Oxoid, Basingstoke, UK), supplemented with 5% (v/v) sterile horse blood (Oxoid), 5.0 mg/mL hemin (Sigma, St. Louis, MO, USA) and 1.0 mg/mL menadione (Merck, Darmstadt, Germany) in anaerobic conditions (10% H2, 10% CO2, and balance N2) at 37ºC for 24-72 h.

-Saliva Preparation

Un-stimulated saliva was obtained from healthy volunteers in 10 ml aliquots at least 1.5 h after eating, drinking or tooth brushing. Saliva preparation were carried out as previously described (20). The efficacy of this protocol was assessed by plating processed saliva samples onto supplemented blood agar plates for 72 h at 37ºC and confirmed by the lack of any bacterial growth on either aerobically or anaerobically incubated plates.

-Biofilm development assays

Biofilms were developed as previously described (20). In brief, pure cultures were grown anaerobically in a protein rich medium containing brain-heart infusion (BHI) (Becton, Dickinson and Company, USA) supplemented with 2.5 g/L mucin (Oxoid), 1.0 g/L yeast extract (Oxoid), 0.1 g/L cysteine (Sigma), 2.0 g/L sodium bicarbonate (Merck), 5.0 mg/mL hemin (Sigma), 1.0 mg/mL menadione (Merck) and 0.25% (v/v) glutamic acid (Sigma). The bacterial growth was adjusted by spectrophotometry to mid-exponential phase with the objective to obtain a solution in modified BHI medium containing 103 colony forming units (CFU)/mL for S. oralis, 105 CFU/mL for *V. parvula* and *A. naeslundii*, and 106 CFU/mL for *F. nucleatum, A. actinomycetemcomitans* and *P. gingivalis*. Sterile calcium hydroxy-apatite disks of 7 mm of diameter and 1.8 (SD=0.2) mm of thickness (Clarkson Chromatography Products, Williamsport, PA, USA) were coated with treated saliva for 4 h at 37ºC in sterile plastic tubes, and then placed in the wells of a 24-well tissue culture plate (Greiner Bio-one, Frickenhausen, Germany). Each well was inoculated with 1.5 mL pooled bacteria culture prepared and incubated in anaerobic conditions (10% H2, 10% CO2, and balance N2) at 37ºC for 72 h. The plates employed for assessing the sterility of the culture medium were used as controls.

-Tested mouthrinses

The following commercially available mouthrinses were evaluated: Perioaid treatment® (Dentaid, Cerdanyola, Spain) containing 0.12% CHX and 0.05% CPC as active ingredients, without alcohol (CHX/CPC); Listerine® (Johnson & Johnson, Madrid, Spain) containing a combination of four essential oils (EO) as active ingredients (thymol 0.06%, eucalyptol 0.09%, methyl salicylate 0.06%, menthol 0.01%) in an alcoholic solution; and Meridol® (GABA GmbH, Lorrach, Switzerland), containing amine fluoride/stannous fluoride (AFSF) without alcohol. PBS and absolute ethanol (EtOH) served as the negative and positive controls, respectively.

-Exposure to oral rinses

To evaluate the bactericidal action of CHX/CPC, EO and AFSF wells containing 2 mL of suspension of the tested products, EtOH and PBS were prepared and once the 72-h biofilms were formed over HA discs they were transferred to the wells. Following a single 1 minexposure, discs were sequentially washed three times in 2 mL of fresh PBS (immersion time per rinse, 10 s).

In each experiment, the three mouthrinses and the control solutions were tested together. The experiments were repeated nine times, on different days and with fresh bacterial cultures.

-Cell viability assessed by ATP bioluminescence

Immediately after treatment, each HA disc was transferred to sterile plastic tubes containing 1.0 mL of PBS, and vortexed vigorously for 2 min, to harvest adherent cells. The BacTiter-Glo viability assay kit® (Promega, Madison, WI, USA) was utilized to assess the ATP bioluminescence, following the manufacturer´s instructions. This assay is based on the luciferase-catalysed reaction of luciferin and ATP and thus it quantifies the ATP present signalling the presence of metabolically active cells. In brief, 400 µL of BacTiter-Glo reagent was added with gentle stirring to a plastic cuvette containing 400 µL of disaggregated biofilm cells and incubated in darkness for 5 min at room temperature. The luminescent signal was recorded for 1 s per cuvette into a 1250 Luminometer (LKB-Wallac, Turku, Finland). The light output and ATP measurements were done at room temperature and expressed in Relative Light Units (RLUs) per mL. The culture medium and PBS, in the absence of cells, served as negative controls since they contained no detectable ATP.

-Cell viability assessed by culture methods

Using the same biofilms (as described in the previous section), standard microbiologic culture methods were carried out and the number of obtained colonies was compared with the ATP bioluminescence results. In brief, the disaggregated biofilms were subjected to 10-fold serial dilutions in PBS and then plated onto blood agar plates (Blood Agar Oxoid No 2), supplemented with 5% (v/v) sterile horse blood (Oxoid), 5.0 mg/mL hemin (Sigma) and 1.0 mg/mL menadione (Merck) in anaerobic conditions (10% H2, 10% CO2, and balance N2) at 37ºC. All plating procedures were conducted in duplicate, and the number of colonies (between 30 and 300) were used to calculate the number of viable bacteria from a particular dilution and averaged to determine mean values colony forming units (CFU/mL).

-Statistical analysis

Data were calculated as RLU/mL, for samples analyzed by ATP bioluminescence, and as CFU/mL, for samples analyzed by culture-method. The proportions of vital cells after mouthrinse contact, as compared to the negative control, were calculated for both techniques. The vitality ratio was calculated by dividing the vitality value for each mouthrinse to the one given by the negative control.

An experiment-level analysis was performed for each study parameter (n=9). Kolmogorov-Smirnov goodness-of-fit tests were computed for each variable. Data were expressed as means and standard deviations (SD). Box-plots were used for the graphic presentation of data. To test the effect of each mouthrinse on cell vitality, analysis of variance (ANOVA) and post hoc-testing with Bonferroni´s correction for multiple comparisons were used for both techniques (mean viable cell count as CFU/mL, and the ATP-driven bioluminescence determinations, as RLU/mL). To determine the reliability of ATP-driven bioluminescence to measure cell vitality, Pearson correlation coefficient was determined between the ratio of vitality obtained by culture and ATP-driven bioluminescence. In addition, the existence of differences between ratios of vitality obtained by each technique was assessed by the Student t-test for independent samples.

Results were considered statistically significant at p<0.05. A software package (*IBM® SPSS®* Statistics 19.0) was used for all data analysis.

## Results

-ATP-driven bioluminescence

**Figure 1:**
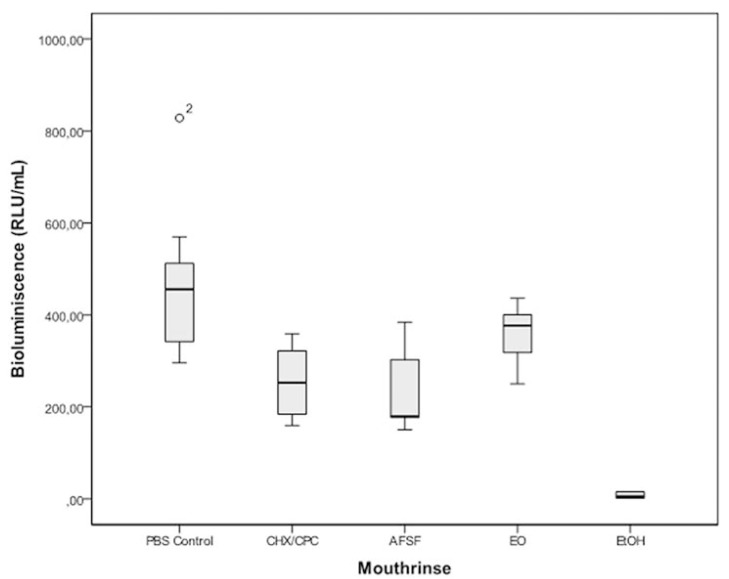
Box plots showing ATP bioluminescence values from in vitro subgingival biofilms after treatment with the mouthrinses containing chlorhexidine digluconate and cetylpyridinium chloride (CHX/CPC), amine fluoride/stannous fluoride (AFSF) and essential oils (EO) compared to a negative PBS control and a positive EtOH control (n = 9). Differences between control and CHX/CPC and AFSF treatments were statistically significant (p<0.005). Differences between treatments were not significant. (RLU: Relative Light Units).

All tested mouthrinses reduced the amounts of ATP-driven bioluminescence, as compared to the negative control solution. Statistically significant differences in bioluminescence were observed for CHX/CPC (mean difference 217.08 RLU/mL, 95% confidence interval -CI- [87.84, 346.32]; p<0.001) and AFSF treatment (243.69, CI [114.45, 372.93]; p<0.001) when compared to the negative control. No statistically significant changes in bioluminescence were found for EO when compared to the negative control (119.79, CI [-9.45, 249.04]; p=0.088). No statistically significant differences were found when the mouthrinses where compared. (Fig. 1) presents the comparative effect of CHX/CPC, EO and AFSF mouthrinses on the cell viability of biofilms assessed by ATP bioluminescence (RLU/mL). The lowest values were found for AFSF (233.08; SD=83.13), followed by CHX/CPC (259.69; SD=78.29), EO (356.98; SD=63.01) and the negative control (476.78; SD=53.25). AFSF, however, demonstrated the largest variability in the response.

-Culture-dependent method

**Figure 2:**
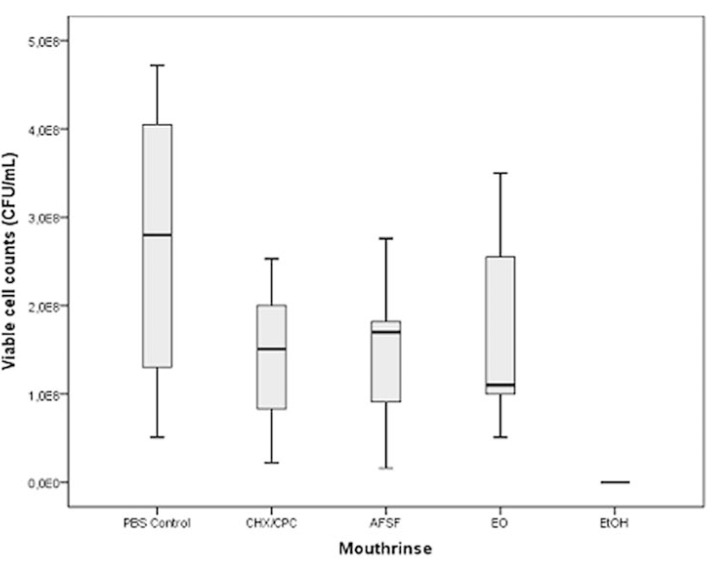
Box plots showing viable cell count as Colony-Forming Units per mL values from in vitro subgingival biofilms after treatment with the mouthrinses containing chlorhexidine digluconate and cetylpyridinium chloride (CHX/CPC), amine fluoride/stannous fluoride (AFSF) and essential oils (EO) compared to a negative PBS control and a positive EtOH control (n = 9). Differences between control and all treatments were not significant. Differences among treatments were not significant.

Figure 2 depicts the effect of the tested mouthrinses over the biofilms, as assessed by viable cell counts obtained by standard culturing (CFU/mL). The lowest cell viability values were found for CHX/CPC (1.38x108 CFU/mL; SD=8.54x107), followed by AFSF (1.42x108; SD=9.03 x107), EO (1.67x108; SD=1.17x108) and the negative control (2.55x108; SD=1.63x108). All groups presented large interquartile-range variability, being the largest deviations from the obtained median values shown by AFSF and EO. No statistically significant differences in viability cell counts were observed among mouthrinses or between each mouthrinse and the negative control.

-Comparison between ATP-driven bioluminescence and culture-dependent method

 Table 1 shows the comparisons between obtained ratios of vitality by ATP bioluminescence and by culture. A statistically significant correlation was observed (r=0.769, p<0.001) between both techniques. No statistically significant differences were found between the ratios of vitality for each mouthwash, when calculated by either technique.

**Table 1:**
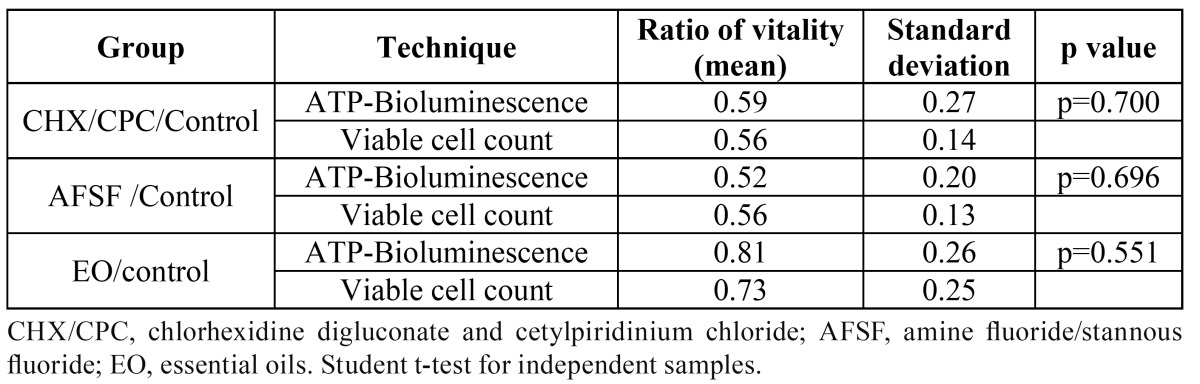
Ratios of vitality obtained with each mouthrinse by ATP-Bioluminescence and viable cell count.

## Discussion

The objective of this study was to validate the use of the ATP bioluminescence method for rapid quantitative evaluation of viable total oral bacteria in biofilm samples after exposure to the antimicrobial agents. The obtained results have shown that the ATP bioluminescence method was capable of quantifying bacterial vitality, when applied to a validated *in vitro* biofilm model. In fact the relative antimicrobial effect of three tested antimicrobial compounds was demonstrated with this method when compared with a negative control and these results were similar when compared with standard culturing techniques.

The exposure of a relatively mature biofilm to a 1-min contact with the tested antimicrobials resulted in a bactericidal effect, demonstrating a significant reduction in the viable microbial load when compared to the negative control. EO was substantially less effective than CHX/CPC and AFSF, which depicted similar antimicrobial activity, although AFSF demonstrated a higher variability in its antimicrobial effect. CHX/CPC, therefore, demonstrated the most efficacious activity in this *in vitro* biofilm model. These results are in agreement with the reported significant antimicrobial effect of a CHX formulation, as compared to a control, when evaluated by ATP bioluminescence in *P. gingivalis* biofilms (21), as well as with those reported in other studies using different biofilm models (9,10,11,22). All these experiments reported that the CHX-containing mouthrinses had significantly higher bactericidal activity than EO- and AFSF-containing formulations. Multiple long-term home-use randomized clinical trials have also demonstrated that CHX-based products are the most effective against plaque and gingivitis, with demonstrated antiplaque and antigingivitis effect (23). In addition, CHX-formulations have demonstrated better clinical results when compared to EO- and AFSF-formulations (24,25).

The microbiological assay used in this study was the ATP bioluminescence technique. This method has previously shown valid to provide a real-time estimation of total viable bacteria in biological sample (19), although not tested before with a biofilm containing subgingival bacteria. Prior studies have shown a correlation between ATP measurements and the viable bacterial number obtained by standard culturing techniques (17,26,27) for the determination of total oral bacteria. The data from this investigation also showed a statistically significant correlation (Pearson correlation coefficient of 0.769, p<0.001) between both techniques. In addition, no statistically significant differences between the relative proportions of cell vitality values for each mouthwash calculated with both techniques were observed. These results confirm, therefore, the ability of this ATP technology as a suitable method to evaluate bacterial viability in an oral biofilm model, since the luminescent signal generated during cell lyses was proportional to the amount of ATP present. This technique has also being recently applied in other oral investigations, such as: the study of different treatment approaches on the removal of early plaque biofilms grown on titanium implants (16), the detection of cariogenic bacteria genes by a combination of allele-specific polymerase chain reactions and a novel bioluminescent pyrophosphate assay (15) and in a randomized clinical study of plaque retention by self-ligating versus elastomeric orthodontic brackets (17).

In conclusion, the results of the present study have validated the ATP bioluminescence method for evaluating the bacterial viability in an *in vitro* biofilm model. It has also been demonstrated the utility of this *in vitro* biofilm model to test the antimicrobial effect of mouthrinses.
